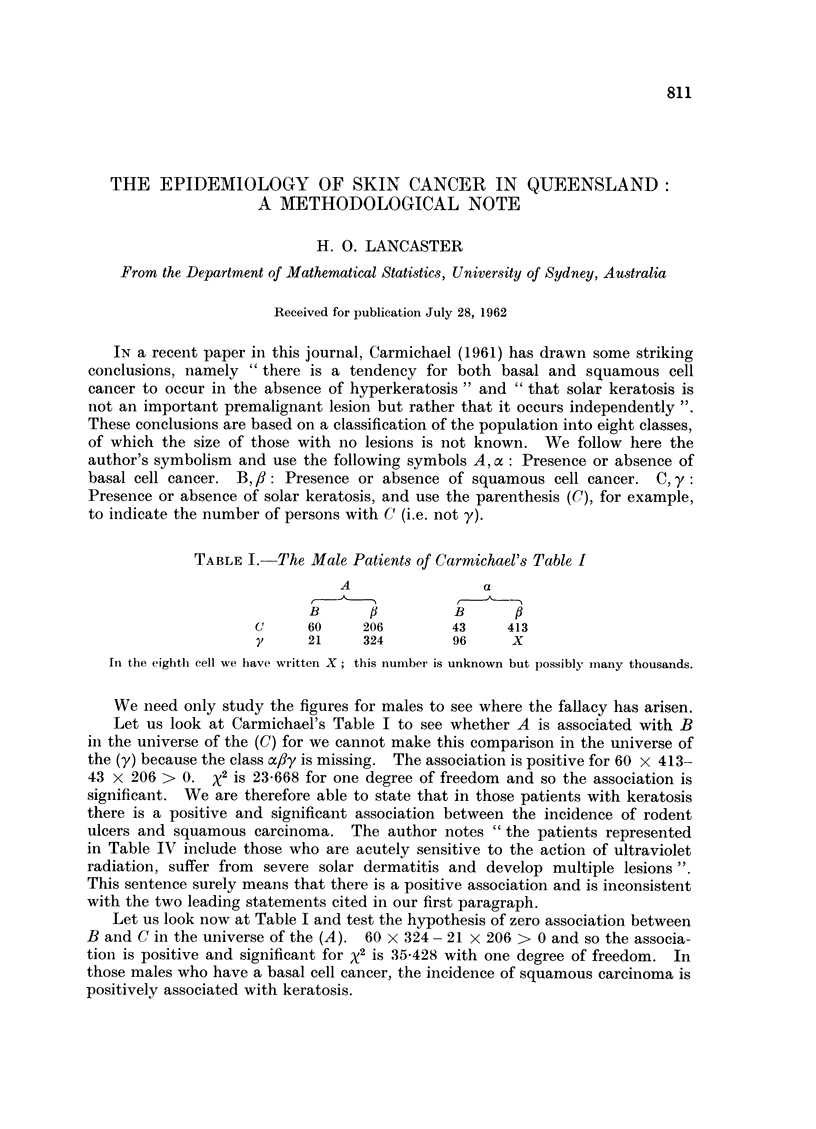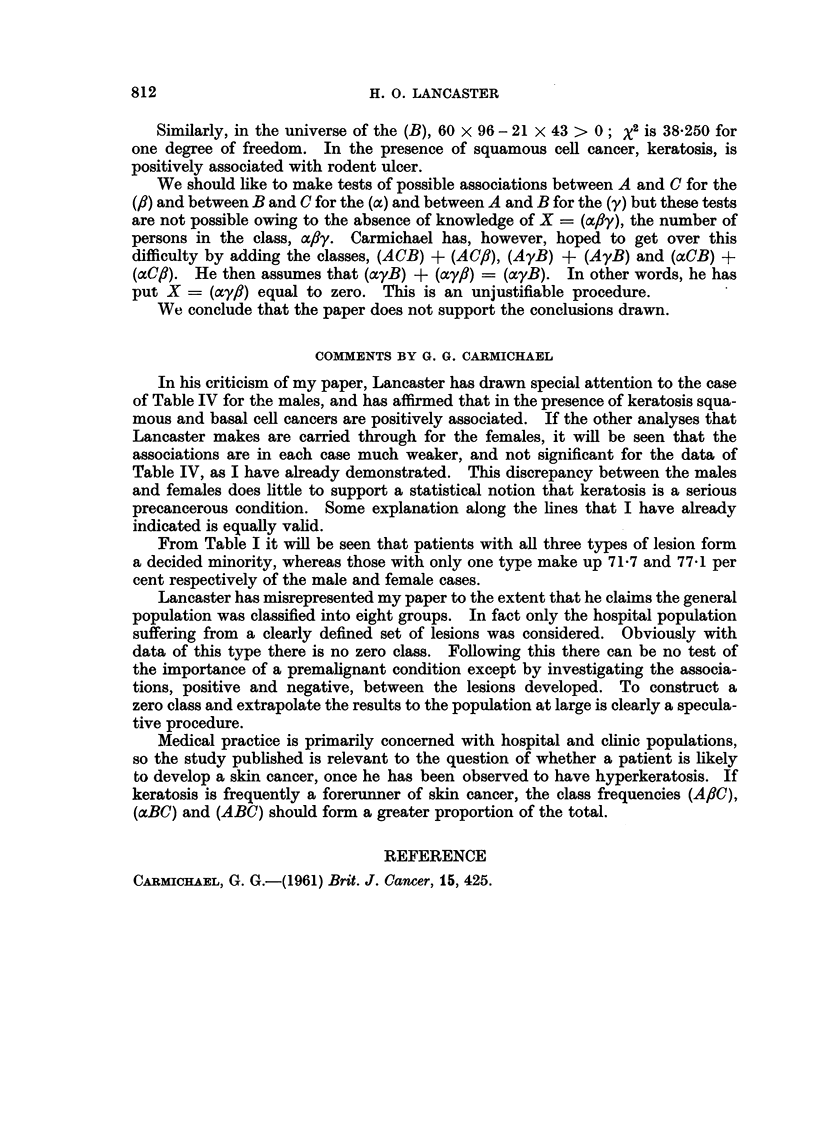# The Epidemiology of Skin Cancer in Queensland: A Methodological Note

**DOI:** 10.1038/bjc.1962.94

**Published:** 1962-12

**Authors:** H. O. Lancaster


### THE EPIDEMIOLOGY OF SKIN CANCER IN QUEENSLAND: A METHODOLOGICAL NOTE

H. O. LANCASTER

*From the Department of Mathematical Statistics, University of Sydney, Australia*

Received for publication July 28, 1962

In a recent paper in this journal, Carmichael (1961) has drawn some striking conclusions, namely " there is a tendency for both basal and squamous cell cancer to occur in the absence of hyperkeratosis " and " that solar keratosis is not an important premalignant lesion but rather that it occurs independently ". These conclusions are based on a classification of the population into eight classes, of which the size of those with no lesions is not known. We follow here the author's symbolism and use the following symbols $A, \alpha$ : Presence or absence of basal cell cancer. B, $\beta$ : Presence or absence of squamous cell cancer. C, $\gamma$ : Presence or absence of solar keratosis, and use the parenthesis ($C$), for example, to indicate the number of persons with $C$ (i.e. not $\gamma$).

TABLE I.—*The Male Patients of Carmichael's Table I*

| | $A$ | | $\alpha$ | |
|---|---|---|---|---|
| | $B$ | $\beta$ | $B$ | $\beta$ |
| $C$ | 60 | 206 | 43 | 413 |
| $\gamma$ | 21 | 324 | 96 | $X$ |

In the eighth cell we have written $X$; this number is unknown but possibly many thousands.

We need only study the figures for males to see where the fallacy has arisen.

Let us look at Carmichael's Table I to see whether $A$ is associated with $B$ in the universe of the ($C$) for we cannot make this comparison in the universe of the ($\gamma$) because the class $\alpha\beta\gamma$ is missing. The association is positive for $60 \times 413 - 43 \times 206 > 0$. $\chi^2$ is 23·668 for one degree of freedom and so the association is significant. We are therefore able to state that in those patients with keratosis there is a positive and significant association between the incidence of rodent ulcers and squamous carcinoma. The author notes " the patients represented in Table IV include those who are acutely sensitive to the action of ultraviolet radiation, suffer from severe solar dermatitis and develop multiple lesions ". This sentence surely means that there is a positive association and is inconsistent with the two leading statements cited in our first paragraph.

Let us look now at Table I and test the hypothesis of zero association between $B$ and $C$ in the universe of the ($A$). $60 \times 324 - 21 \times 206 > 0$ and so the association is positive and significant for $\chi^2$ is 35·428 with one degree of freedom. In those males who have a basal cell cancer, the incidence of squamous carcinoma is positively associated with keratosis.

Similarly, in the universe of the $(B)$, $60 \times 96 - 21 \times 43 > 0$ ; $\chi^2$ is 38·250 for one degree of freedom. In the presence of squamous cell cancer, keratosis, is positively associated with rodent ulcer.

We should like to make tests of possible associations between $A$ and $C$ for the $(\beta)$ and between $B$ and $C$ for the $(\alpha)$ and between $A$ and $B$ for the $(\gamma)$ but these tests are not possible owing to the absence of knowledge of $X = (\alpha\beta\gamma)$, the number of persons in the class, $\alpha\beta\gamma$. Carmichael has, however, hoped to get over this difficulty by adding the classes, $(ACB) + (AC\beta)$, $(A\gamma B) + (A\gamma B)$ and $(\alpha CB) + (\alpha C\beta)$. He then assumes that $(\alpha\gamma B) + (\alpha\gamma\beta) = (\alpha\gamma B)$. In other words, he has put $X = (\alpha\gamma\beta)$ equal to zero. This is an unjustifiable procedure.

We conclude that the paper does not support the conclusions drawn.

### COMMENTS BY G. G. CARMICHAEL

In his criticism of my paper, Lancaster has drawn special attention to the case of Table IV for the males, and has affirmed that in the presence of keratosis squamous and basal cell cancers are positively associated. If the other analyses that Lancaster makes are carried through for the females, it will be seen that the associations are in each case much weaker, and not significant for the data of Table IV, as I have already demonstrated. This discrepancy between the males and females does little to support a statistical notion that keratosis is a serious precancerous condition. Some explanation along the lines that I have already indicated is equally valid.

From Table I it will be seen that patients with all three types of lesion form a decided minority, whereas those with only one type make up 71·7 and 77·1 per cent respectively of the male and female cases.

Lancaster has misrepresented my paper to the extent that he claims the general population was classified into eight groups. In fact only the hospital population suffering from a clearly defined set of lesions was considered. Obviously with data of this type there is no zero class. Following this there can be no test of the importance of a premalignant condition except by investigating the associations, positive and negative, between the lesions developed. To construct a zero class and extrapolate the results to the population at large is clearly a speculative procedure.

Medical practice is primarily concerned with hospital and clinic populations, so the study published is relevant to the question of whether a patient is likely to develop a skin cancer, once he has been observed to have hyperkeratosis. If keratosis is frequently a forerunner of skin cancer, the class frequencies $(A\beta C)$, $(\alpha BC)$ and $(ABC)$ should form a greater proportion of the total.

### REFERENCE

Carmichael, G. G.—(1961) *Brit. J. Cancer*, **15**, 425.